# IL-10 secreting regulatory B cells are potent arbiters of autoimmunity in both mouse and man

**DOI:** 10.1186/1479-5876-9-S2-I12

**Published:** 2011-11-23

**Authors:** Claudia Mauri, Fabian Flores-Borja, Paul A Blair, Natalie A Carter

**Affiliations:** 1 University College London, Division of Medicine, Centre for Rheumatology Research, The Rayne Institute, London, UK; 2 Medical Research Council Centre for Transplantation, Dept. of Nephrology and Transplantation, Guy's Hospital, Kings College London, London, UK

## Background

The existence of B cells with a suppressive function, namely regulatory B cells (Bregs), has been demonstrated in a number of murine models of autoimmunity in the last decade. Adoptive transfer of Bregs has been shown to inhibit the development of several autoimmune diseases via the release of Interleukin-10 (IL-10).

## Aim

To ascertain the role of IL-10 secreting Bregs during the induction and progression of experimental autoimmune disease in mice.Then to use this information to identify an equivalent subset in man and assess the impact of Bregs on inflammatory responses and disease severity.

## Materials and methods

We generated chimeric mice that lack IL-10 specifically in their B cells and used this model to investigate the role of endogenous B cell- derived IL-10 during inflammatory arthritis. Moreover, we have identified and assessed the role of Bregs in healthy individuals as well as patients with rheumatological disorders.

## Results

We have recently investigated the mechanisms utilized by endogenous Breg at the cellular level in an experimental model of arthritis and shown that the IL-10 produced by Bregs is important in the maintenance of regulatory T cells (Tregs) whilst suppressing Th1 and Th17 responses. Indeed chimeric mice specifically lacking IL-10 producing B cells (IL-10^-/-^B cell) developed an exacerbated arthritis, displayed reduced absolute numbers of FoxP3+ Tregs, and a marked increase in inflammatory Th1 and Th17 cells compared to chimeric wild type B cell mice. In addition we extended our results from experimental models of arthritis to humans and identified an equivalent population of Breg that produce IL-10, inhibit CD4^+^T effector responses and convert effector CD4+T cells into regulatory T cells.

## Conclusions

Our work has demonstrated the importance of Bregs in controlling inflammatory responses and preventing autoimmunity in both experimental arthritis in mice [1,2] and patients with rheumatological disorders [3]. This presentation reports the function of this pivotal B cell subset and describes Bregs in the context of healthy individuals as well as in patients with systemic lupus erythematosus and rheumatoid arthritis.The impact of altered cellular function within the Breg compartment on disease outcome will be also discussed [4,5].
